# Assessing the Quality of Whole Genome Alignments in Bacteria

**DOI:** 10.1155/2009/749027

**Published:** 2009-11-15

**Authors:** Firas Swidan, Ron Shamir

**Affiliations:** The Blavatnik School of Computer Science, Tel Aviv University, Tel Aviv 69978, Israel

## Abstract

Comparing genomes is an essential preliminary step to solve many problems in biology. Matching long similar segments between two genomes is a precondition for their evolutionary, genetic, and genome rearrangement analyses. Though various comparison methods have been developed in recent years, a quantitative assessment of their performance is lacking. Here, we describe two families of assessment measures whose purpose is to evaluate bacteria-oriented comparison tools. The first measure is based on how well the genome segmentation fits the gene annotation of the studied organisms; the second uses the number of segments created by the segmentation and the percentage of the two genomes that are conserved. The effectiveness of the two measures is demonstrated by applying them to the results of genome comparison tools obtained on 41 pairs of bacterial species. Despite the difference in the nature of the two types of measurements, both show consistent results, providing insights into the subtle differences between the mapping tools.

## 1. Introduction

**Figure 1:**
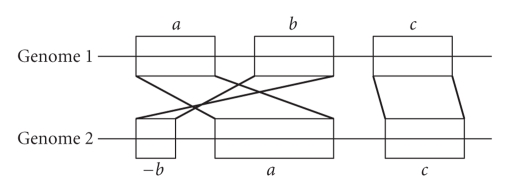
A schematic illustration of a genome mapping result. Each genome (illustrated as a straight line) is broken into segments or fragments (the labeled rectangular blocks), and each segment is mapped to a corresponding one in the other genome. The breakage of the genome into segments is referred to as *segmentation*. Segments with an identical label but different signs have reversed orientation. Note that a segmentation may leave regions outside of the blocks. Those regions are considered segmental insertions/deletions and are not included in the mapping between the genomes.

With the dramatic increase in the number of sequenced genomes, comparative genome analysis has become increasingly common. Evolutionary, genetic, and genome rearrangement studies require as a first step the comparison of two whole genomes, referred to as *genome mapping* or *whole genome alignment*, with subsequent analyses dependent on the quality of the mapping [1–3]. This mapping (Figure 1) usually consists of a segmentation of the two genomes into fragments, and the matching of fragments between the two genomes according to type of evolutionary relations: for example, orthology, paralogy, segmental deletions and insertions (*segmental indels*), or rearrangement [4–6].

Though numerous mapping procedures have been proposed in recent years, few objective criteria have been suggested for quantitatively assessing them. Early methods relied mainly on gene annotation for building the mappings: for example, [7] was based on P-quasi grouping, [8–10] tried to identify contiguity and gene clusters, [11] used an alignment-like approach, and [12] relied on gene correspondence. Later methods utilized the increase in the available genomic sequences resulting from advances in genome assembly techniques [13]: examples are BlastZ [14] which was applied on the mouse and human genomes [15], and the genome-rearrangement approaches in [16–19]. Methods that addressed bacterial genomes include Mauve [20], its predecessor GRIL [21], MAGIC [22] (see Section 2 for details), and [23]. Methods were recently developed to evaluate the accuracy of alignments on the whole genome level [2]. In contrast, we describe two methods that evaluate the quality of the mapping as a whole, including its induced segmentation of the genomes and the inferred relations between the resulting fragments (Figure 1).

We present two families of simple, biologically intuitive measures for quantitatively assessing the quality of bacterial genome mapping. The first family relies on the assumption that evolutionary changes are not likely to disrupt genes; hence, a better genome mapping should have fewer gene disruptions and its induced segmentation should show a better fit to known gene annotations. The second family uses two factors: segmentation size (the number of fragments in the mapping), and whole genome conserved percentage (the number of exact base matches in the global alignment of the mapping's induced fragments divided by the genome size). Clearly, a mapping with more fragments will have a higher conserved percentage.

The power of the measures is demonstrated by applying them to the results of the genome mapping tools MAGIC [22] and Mauve [20] on 41 pairs of bacterial species. Both MAGIC and Mauve were designed specifically for bacterial genome mapping. (Tools designed for eukaryotic genome mapping—for example, CHAIN-NET [15], FISH [24], GRIMM-Synteny [19], SLAGAN [16], TBA [25], and the more recent approach of [18]—are geared toward handling much larger genomes possessing extremely different characteristics.) As we will show, the measures are capable of discriminating between the results of the mapping tools, and providing quantitative estimates on their performance.

## 2. Materials and Methods

### 2.1. Mapping Tools

The following is an overview of MAGIC and Mauve. Detailed descriptions are given in [20, 22] and on the websites http://magicmapping.sourceforge.net/ for MAGIC and http://gel.ahabs.wisc.edu/mauve for Mauve. MAGIC C/C++ implementation version 1.0 and Mauve version 2.2.0 were used (the most recent versions at the time the comparisons were performed).

*A brief sketch of the two algorithms.* Procedures for mapping between genomes can be conceptually divided into two phases: a pre-processing phase aimed at finding maximal local similarities between the genomes, and a mapping phase aimed at inferring one-to-one correspondences out of these similarities. In MAGIC, a linear pipeline of global and local alignments is used to compute a comprehensive set of maximal similar regions in the two genomes. This phase is initialized with a set of anchors between the two genomes. Normally, MAGIC uses annotated genes as anchors, but here anchors were provided from Mauve (see below) in order to avoid bias in the assessment scores. The result of the pre-processing phase is then iteratively clustered into reorder-free (RF) regions, while resolving conflicts between its different entries based on contextual hints. In Mauve, the pre-processing phase calculates maximal unique matches (MUMs) and filters them according to their lengths. Then, in the mapping phase, overlaps between the MUMs are resolved in a pairwise fashion, and locally collinear blocks (LCBs) are calculated based on iterative breakpoint analysis. Out of the final set of LCBs, Mauve calculates its backbones—one-to-one LCB correspondences containing no big gaps. These backbones were used here also as anchors for MAGIC.

MAGIC and Mauve use different approaches to filtering mobile DNA elements or *mobilome* [26, 27]. Mobile DNA content is high in some of the compared bacteria, reaching up to 20% (e.g., in *Streptococcus pyogenes* [22]). To make handling mobile DNA as comparable as possible, MAGIC's mobile DNA filtering step was deactivated, and its length threshold for discarding entries at the beginning of the mapping phase was increased from 200 bp to 1000 bp (see [22] for details). This change is disadvantageous to MAGIC as it forces it to deviate from the native settings. In addition, both MAGIC and Mauve were run with their default parameters. On average, MAGIC's run takes 118 seconds, while Mauve's takes 35 seconds.

### 2.2. Gene Annotation and Gene Disruptions (GDs)

Gene annotations for the different prokaryotic organisms are obtained through KEGG (Kyoto Encyclopedia of Genes and Genomes) [28].

To reduce the sensitivity of the GD scores to gene end annotation errors, we counted a breakpoint induced from the mapping as disrupting a gene only if it was located inside the gene and at a considerable distance (>10% of the gene′s length) from its end.

### 2.3. Conserved Percentages

The end result of MAGIC and Mauve is a mapping between rearrangement-free segments in one genome to their counterparts in the second genome. To calculate conserved percentages for our purposes, these segments were globally aligned in a post-processing step and orthologous segments were identified using the procedure for calculating backbones described in [20]. The conserved percentage was defined as the number of base matches in these fragments divided by the size of the genome (Section 3.2).

### 2.4. Statistical Tests

The differences observed in the measures defined in Section 3 are quantified by two statistical tests: the one-sided sign (binomial) test [29] and the one-sided Wilcoxon signed-rank test [30] (see also [31] for more details). The null hypothesis for these tests states that the results of Mauve are at least as good as those of MAGIC. The significance level is set to 0.05 for both tests.

## 3. Results

We present each measure and its results. More details about the mapping tools, gene annotations, and statistical tests can be found in Section 2.

### 3.1. Gene Annotation-Based Measure

Given a mapping between two genomes, we used the induced segmentation in each of the genomes to evaluate the quality of the mapping. Each of the two segmentations was assigned a *Gene Disruption (GD) score* denoting how many genes the segmentation disrupts. We say that a segmentation disrupts a gene if it has a segment end within the gene that is sufficiently far from the gene's ends (see Section 2.2). The GD-score of the mapping is defined as the sum of the GD-scores of both segmentations.

**Figure 2:**
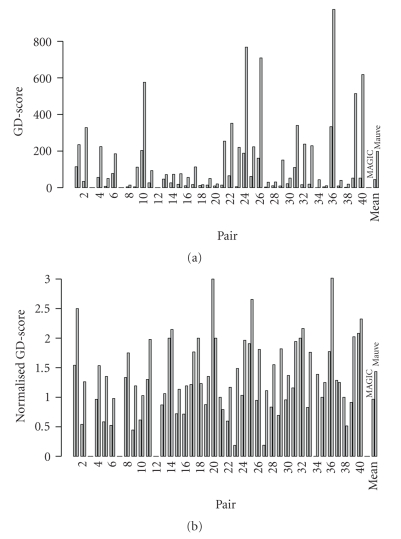
(a) GD-scores and (b) their normalization by segmentation sizes for both MAGIC and Mauve. The *X*-axis lists the pairs (as in Table 1), with MAGIC and Mauve results represented in the left and right bars, respectively, of each pair. The *Y*-axis is the scores. The rightmost column is the mean score for the method.

The GD-scores for the 41 pairs (Table 1) are summarized in Figure 2(a). In 37 pairs, either MAGIC or Mauve was assigned a non-zero GD-score. MAGIC did not disrupt any genes in five pairs, and Mauve in four. MAGIC's GD-scores ranged from 0 to 333, while Mauve's GD-scores ranged from 0 to 974. MAGIC's score was lower than or equal to Mauve's on all pairs: MAGIC had lower scores in 37 pairs, and in the four remaining pairs both methods were assigned a score of 0. The results were significantly in favor of MAGIC (*P*-value 7 × 10^−12^; sign test). The mean GD-score values were 44 and 198 for MAGIC and Mauve, respectively; the difference is statistically significant (*P*-value 6 × 10^−8^; Wilcoxon test).

#### 3.1.1. Dependency of Scores on Segmentation Size

Because a mapping with more segments can disrupt more genes, we analyzed the GD-score dependency on the segmentation size (the number of fragments in the mapping) by first normalizing the score according to the segmentation size, and then linearly fitting the score to the segmentation size.

Figure 2(b) presents the results for the normalization. Here, the scores were divided by the segmentation size. The normalized GD-scores of Magic and Mauve ranged from 0 to 3. (The maximum possible value of 4 occurs if two mapped segments disrupt two genes in each of the genomes.) MAGIC had lower normalized scores in 32 pairs, while Mauve had lower ones in 5 pairs. MAGIC's advantage was significant (*P*-value 4 × 10^−6^, sign test). The mean normalized GD-scores of MAGIC and Mauve were 1 and 1.4, respectively; the difference is significant (*P*-value 4 × 10^−6^, Wilcoxon test).

**Figure 3:**
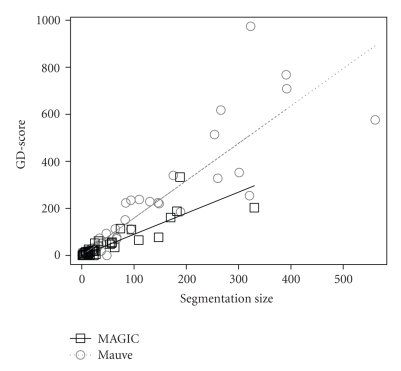
The GD-scores as a function of segmentation size for MAGIC and Mauve. The *X*-axis is the segmentation size. The *Y*-axis is the GD-scores. Least-squares estimated linear fittings are shown as straight lines.

Figure 3 shows a linear fitting of the scores to the segmentation sizes. The linear fitting is constrained to pass through the origin (0 segments imply 0 score). The estimated slopes are 0.9 ± 0.07 and 1.6 ± 0.1 for MAGIC and Mauve, respectively (*R* ^2^ = .82 and .86, resp.).

### 3.2. Segmentation-Matching Based Measure

 This measure assesses the coverage of the mapping and its accuracy at the single base level. The *conserved percentage* of a genome is defined as the number of exact base matches in the segments' alignments—as dictated by the mapping—divided by the genome size. The *conserved percentage* of a pair of genomes is the mean of their conserved percentages. Increasing the number of segments in both genomes allows more freedom in the correspondence between them, which can improve their conserved percentage. Hence, a mapping with both a smaller segmentation size and a greater conserved percentage is deemed superior.

**Figure 4:**
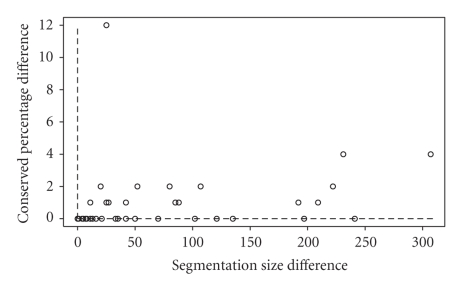
The difference between Mauve and MAGIC in segmentation size and matching percentage.

Table 1 gives the segmentation sizes and conserved percentages obtained for MAGIC and Mauve. Figure 4 plots, for each pair, the difference between the values of Mauve and MAGIC for the two criteria. The average MAGIC and Mauve segmentation sizes were 46 and 122 respectively. There are 22 pairs on which MAGIC dominates Mauve, and one pair (#3) on which both methods reported equal results. On the rest of the pairs, MAGIC reported a smaller segmentation size and a smaller conserved percentage. On these pairs, the difference in the segmentation size could be as high as 307 in favor of MAGIC (average of 78). The difference in conserved percentages, on the other hand, could be as high as 12% in favor of Mauve (average of 2%).

**Figure 5:**
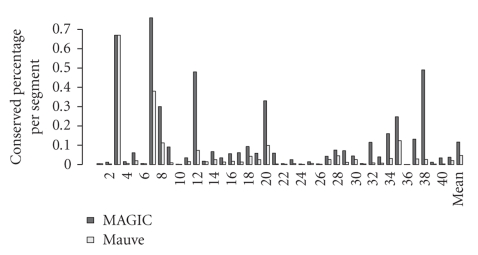
The conserved percentage divided by the segmentation size.

Figure 5 gives the conserved percentages divided by the segmentation sizes. MAGIC's ratios were greater than Mauve's in 40 pairs, a statistically significant result (*P*-value 2 × 10^−11^, sign test). Their means were .12 and .05, respectively, a significant difference (*P*-value 1 × 10^−9^; Wilcoxon test).

Finally, to further investigate cases where neither method dominated the other, we artificially constrained MAGIC to output a mapping as close in size as possible to Mauve's on the 18 nonconclusive pairs. In this exercise, MAGIC dominated Mauve on 5 pairs and Mauve dominates MAGIC on 2 pairs (out of the 18 nonconclusive pairs). In total, MAGIC dominated Mauve on 27 pairs (along with the previous 22 pairs), a result that is statistically significant (*P*-value 0.02, sign test).

## 4. Discussion

We presented two types of measures for assessing genome mapping results. Both are very simple, biologically intuitive, and easy to compute. Unlike other evaluation methods that try to estimate the accuracy of genome alignments, such as [2], the criteria presented here aim to provide global measures of the mapping quality. As the results show, the measures consistently discriminate between the two mapping methods that we tested, favoring MAGIC over Mauve.

The GD-score *G* is a function of the segmentation size *S* and the imprecision *I* of the mapping tool: *G* = *f*(*I*, *S*) (the higher the segmentation size or the imprecision, the higher the GD-score). The imprecision *I* is a property of the mapping tool, and is independent of the compared pair. It reflects the tool's tendency to either miscalculate fragment ends or to report erroneous correspondences between segments. The segmentation size *S*, on the other hand, depends on both the tool and the compared pair. Though *G* and *S* are directly measurable, *I* is hidden. Estimating it is possible thanks to the reasonable linear fit between the GD-score and the segmentation size, which implies that *f*(·, ·) can be well approximated with a linear function. The estimation is then carried out by normalizing the GD-score and by linear regression. In general, the GD-score may also be affected by minor gene end annotation errors. This effect, however, was minimized by counting only gene disruptions that occur considerably inside the annotated gene region (Section 2), which also increases the tolerance of the measure to subtle mapping errors near the fragment ends.

Our results on the benchmark set of 41 bacteria pairs suggest that the GD-score is capable of discriminating between the two tools: MAGIC reports lower regular and normalized GD-scores (both with statistical significance) and also has a smaller slope in the linear fit.

The GD-scores linear fitting and the normalization results indicate that MAGIC and Mauve disruption rates (genes disrupted per segment) are below 1.7, compared to a theoretical maximum of 4. Given the high density of genes in bacterial genomes (after the correction discussed in Section 2, genes encompass more than 67% of the studied genomes), the disruption rate under a random model assumption is expected to be greater than 2.6. Thus, the GD-scores indicate that, as expected, the results of both MAGIC and Mauve are better than random.

The segmentation-matching measure provides an additional estimate of the quality of the mapping. Unlike the GD-score, it requires no external information other than the mapping. Yet, like the GD-score, it reflects the imprecision of the mapping tool. Here, inaccuracy in identifying the fragment ends results in a smaller conserved percentage, while an erroneous correspondence between segments increases the segmentation size. For pairs where the measure provides no clear preference for one of the two compared tools, we suggest dividing the conserved percentage by the segmentation size. This ratio reflects the imprecision of the mapping, as it decreases when additional inaccuracies are introduced at fragment ends or when erroneous correspondences are made.

The dominance criterion for this measure, that is, favoring mappings with smaller segmentation size and greater conserved percentage, is fulfilled by MAGIC on 22 pairs. On one pair (#3) both methods report equal results, and on the remaining 18 pairs the results are not conclusive: MAGIC has both smaller segmentation size (a difference of 78 on average) and smaller conserved percentage (2% on average). When the conserved percentage is divided by the segmentation size, MAGIC fares better in 40 out of the 41 pairs (with statistical significance). When MAGIC is constrained to output a mapping of size as close as possible to Mauve's, analysis of the nonconclusive pairs leads to similar conclusions. This observation is notable, since the constraint is expected to be disadvantageous for MAGIC as it forces MAGIC to use an inferior configuration of parameters compared to its default settings.

Since the GD-scores rely explicitly on gene annotations, the evaluated methods should not depend (implicitly or explicitly) on gene annotation for building the genome mapping. For this reason, instead of using gene annotations of KEGG orthologs as seeds in MAGIC, all the above analyses used the Mauve seeds instead. In fact, MAGIC's performance improves according to all the above criteria if its default seeds are used (results not shown). Mauve backbones are fed as initial anchors to MAGIC, further demonstrating that MAGIC's calculated mapping has better quality than its input mapping, in agreement with observations made in [22].

Our main goal here was to define and test some basic measures for evaluating mapping quality. We tested and demonstrated these measures on two mapping tools, but they can be readily used to compare other algorithms. We hope that the availability of established quality measures will advance the important challenge of genome-wide mapping.

## Figures and Tables

**Table 1:** The 41 bacteria pairs. Pair: number of the pair. Organism: names of the organisms. Size: genome size in base pairs. MAGIC: MAGIC's results. Mauve: Mauve's results. Seg.: segmentation size. Ratio: conserved percentage. The pairs are sorted in alphabetical order. For each pair, the best obtained scores are marked in bold.

Pair	Organism	Size	MAGIC		Mauve	
			Seg.	Ratio	Seg.	Ratio
1	*Anaplasma marginale St. Maries*	1197687	**74**	0.35	94	**0.37**
	*Anaplasma phagocytophilum HZ*	1471282				

2	*Bacillus cereus ATCC 14579*	5411809	**63**	**0.77**	260	**0.77**
	*Bacillus cereus E33L (zebra killer)*	5300915				

3	*Blochmannia floridanus*	705557	**1**	**0.67**	**1**	**0.67**
	*Blochmannia pennsylvanicus*	791654				

4	*Bacteroides fragilis YCH46*	5277274	**58**	0.87	146	**0.88**
	*Bacteroides fragilis NCTC 9343*	5205140				

5	*Bartonella henselae Houston-1*	1931047	**12**	0.73	37	**0.74**
	*Bartonella quintana Toulouse*	1581384				

6	*Bordetella pertussis Tohama I*	4086189	**147**	0.79	189	**0.8**
	*Bordetella bronchiseptica RB50*	5339179				

7	*Buchnera aphidicola APS*	640681	**1**	**0.76**	2	**0.76**
	*Buchnera aphidicola Sg*	641454				

8	*Campylobacter jejuni subsp. jejuni NCTC 11168*	1641481	**3**	**0.9**	8	**0.9**
	*Campylobacter jejuni RM1221*	1777831				

9	*Clostridium perfringens 13*	3031430	**9**	0.82	94	**0.83**
	*Clostridium perfringens SM101*	2897393				

10	*Cyanobacteria bacterium Yellowstone A-Prime (Synechococcus sp. JA-3-3Ab)*	2932766	**330**	0.67	561	**0.71**
	*Cyanobacteria bacterium Yellowstone B-Prime (Synechococcus sp. JA-2-3 B*′*a(2-13))*	3046682				

11	*Dehalococcoides ethenogenes 195*	1469720	**20**	0.7	47	**0.71**
	*Dehalococcoides sp. CBDB1*	1395502				

12	*Ehrlichia ruminantium Welgevonden (South Africa)*	1516355	**2**	**0.96**	13	**0.96**
	*Ehrlichia ruminantium Gardel*	1499920				

13	*Francisella tularensis subsp. tularensis SCHU S4*	1892819	**54**	**0.96**	67	**0.96**
	*Francisella tularensis subsp. holarctica OSU18*	1895727				

14	*Haemophilus influenzae Rd KW20 (serotype d)*	1830138	**13**	**0.87**	34	**0.87**
	*Haemophilus influenzae 86-028NP (nontypeable)*	1913428				

15	*Helicobacter pylori 26695*	1667867	**25**	**0.87**	67	**0.87**
	*Helicobacter pylori J99*	1643831				

16	*Listeria monocytogenes EGD-e (serotype 1/2a)*	2944528	**14**	**0.79**	47	**0.79**
	*Listeria innocua CLIP 11262 (serotype 6a)*	3011208				

17	*Legionella pneumophila Lens*	3345687	**14**	**0.86**	64	**0.86**
	*Legionella pneumophila Paris*	3503610				

18	*Mycoplasma genitalium G-37*	580076	**6**	**0.56**	13	**0.56**
	*Mycoplasma pneumoniae M129*	816394				

19	*Mycoplasma hyopneumoniae 232*	892758	**16**	**0.94**	37	**0.94**
	*Mycoplasma hyopneumoniae 7448*	920079				

20	*Mycobacterium tuberculosis H37Rv, laboratory strain*	4411532	**3**	**0.99**	10	**0.99**
	*Mycobacterium tuberculosis CDC1551, clinical strain*	4403837				

21	*Neisseria meningitidis MC58 (serogroup B)*	2272351	**14**	0.83	321	**0.87**
	*Neisseria meningitidis Z2491 (serogroup A)*	2184406				

22	*Nitrobacter winogradskyi Nb-255*	3402093	**109**	0.52	301	**0.53**
	*Nitrobacter hamburgensis X14*	4406967				
23	*Psychrobacter arcticum 273-4*	2650701	**27**	**0.69**	148	**0.69**
	*Psychrobacter cryohalolentis K5*	3059876				

24	*Pseudomonas fluorescens Pf-5*	7074893	**182**	0.54	391	**0.55**
	*Pseudomonas fluorescens PfO-1*	6438405				

25	*Prochlorococcus marinus SS120 (subsp. marinus CCMP1375)*	1751080	**32**	0.46	84	**0.48**
	*Prochlorococcus marinus MIT 9312*	1709204				

26	*Rhodopseudomonas palustris CGA009*	5459213	**170**	0.59	392	**0.61**
	*Rhodopseudomonas palustris HaA2*	5331656				

27	*Rickettsia prowazekii Madrid E*	1111523	**16**	0.69	27	**0.7**
	*Rickettsia felis URRWXCal2*	1485148				

28	*Streptococcus agalactiae 2603 (serotype V)*	2160267	**12**	**0.9**	20	**0.9**
	*Streptococcus agalactiae A909 (serotype Ia)*	2127839				

29	*Staphylococcus aureus subsp. aureus N315, meticillin-resistant (MRSA)*	2814816	**13**	**0.93**	83	**0.93**
	*Staphylococcus aureus subsp. aureus MW2*	2820462				

30	*Shigella flexneri 301 (serotype 2a)*	4607203	**22**	**0.98**	38	**0.98**
	*Shigella flexneri 2457T (serotype 2a)*	4599354				

31	*Staphylococcus haemolyticus JCSC1435*	2685015	**95**	0.49	175	**0.51**
	*Staphylococcus saprophyticus subsp. saprophyticus ATCC 15305*	2516575				

32	*Streptococcus pneumoniae TIGR4*	2160842	**8**	**0.92**	110	**0.92**
	*Streptococcus pneumoniae R6*	2038615				

33	*Streptococcus pyogenes MGAS8232 (serotype M18)*	1895017	**23**	0.91	130	**0.93**
	*Streptococcus pyogenes SSI-1 (serotype M3)*	1894275				

34	*Streptococcus thermophilus CNRZ1066*	1796226	**6**	0.96	31	**0.97**
	*Streptococcus thermophilus LMG18311*	1796846				

35	*Salmonella enterica serovar Typhi CT18*	4809037	**4**	**0.99**	8	**0.99**
	*Salmonella enterica serovar Typhi Ty2*	4791961				

36	*Synechococcus sp. WH 8102*	2434428	**188**	**0.11**	323	**0.11**
	*Synechococcus elongatus PCC6301*	2696255				

37	*Thermus thermophilus HB27*	1894877	**7**	0.92	32	**0.93**
	*Thermus thermophilus HB8*	1849742				

38	*Tropheryma whipplei Twist*	927303	**2**	**0.98**	37	**0.98**
	*Tropheryma whipplei TW08/27*	925938				

39	*Xanthomonas campestris pv. campestris ATCC 33913*	5076188	**57**	**0.68**	254	**0.68**
	*Xanthomonas campestris pv. vesicatoria*	5178466				

40	*Xylella fastidiosa 9a5c*	2679306	**25**	**0.86**	266	**0.86**
	*Xylella fastidiosa Temecula1*	2519802				

41	*Yersinia pestis CO92*	4653728	**23**	0.87	48	**0.99**
	*Yersinia pestis KIM*	4600755				
